# Respiratory syncytial virus, an ongoing medical dilemma: an expert commentary on respiratory syncytial virus prophylactic and therapeutic pharmaceuticals currently in clinical trials

**DOI:** 10.1111/irv.12313

**Published:** 2015-06-09

**Authors:** Lindsay Broadbent, Helen Groves, Michael D Shields, Ultan F Power

**Affiliations:** a Centre for Infection & Immunity, School of Medicine, Dentistry & Biomedical Sciences, Queen's University BelfastBelfast, UK; b The Royal Belfast Hospital for Sick ChildrenBelfast, UK

**Keywords:** respiratory syncytial virus, respiratory syncytial virus antivirals, respiratory syncytial virus prophylactic antibodies, respiratory syncytial virus vaccines

## Abstract

As the most important viral cause of severe respiratory disease in infants and increasing recognition as important in the elderly and immunocompromised, respiratory syncytial virus (RSV) is responsible for a massive health burden worldwide. Prophylactic antibodies were successfully developed against RSV. However, their use is restricted to a small group of infants considered at high risk of severe RSV disease. There is still no specific therapeutics or vaccines to combat RSV. As such, it remains a major unmet medical need for most individuals. The World Health Organisations International Clinical Trials Registry Platform (WHO ICTRP) and PubMed were used to identify and review all RSV vaccine, prophylactic and therapeutic candidates currently in clinical trials. This review presents an expert commentary on all RSV-specific prophylactic and therapeutic candidates that have entered clinical trials since 2008.

## Introduction

Discovered in 1956, respiratory syncytial virus (RSV) was quickly identified as the leading cause of lower respiratory tract infections (LRTI) in infants worldwide.^1^ Respiratory syncytial virus is a member of the Paramyxoviridae family, *Pneumovirus* genus. With a 15·2-kB single-stranded negative sense RNA genome, RSV contains 10 genes encoding 11 proteins, including the fusion (F) and attachment (G) surface glycoproteins, which constitute the principle target antigens for RSV vaccines. Two RSV subgroups exist (A and B), distinguished primarily by genetic and antigenic differences in the G gene and protein. Respiratory syncytial virus virions have two reported forms: spherical particles (≤300 nm diameter) and long filamentous forms (2–10 μm).^2^,^3^ Respiratory syncytial virus is responsible for up to 33·8 million LRTI cases yearly, approximately 3·4 million hospitalisations and up to 199 000 deaths worldwide, predominantly in developing countries.^4^,^5^ For example, Kenya reported RSV-related LRTI rates of 7100/100 000 in children <5 years^6^ versus 1042/100 000 in England.^7^ Furthermore, in many countries, RSV is comparable to influenza regarding mortality rates and health and economic burdens in children.^8^

Symptoms such as rhinorrhea, coryza, sore throat and malaise are features of mild RSV infection.^9^ Clinical signs of RSV-LRTI include dyspnoea, cyanosis, subcostal recession, low-grade fever, wheezing and consolidation.^10^,^11^ RSV-LRTI is responsible for 85% of bronchiolitis and 20% of pneumonia in infants.^12^ In the first year of life, 1–3% of infants are hospitalised with severe RSV-LRTI. Mechanical ventilation is required in 10% of hospitalised infants, of which 5–10% succumb to RSV infection.

Risk factors associated with the development of severe RSV-LRTI include the following: prematurity; bronchopulmonary dysplasia; congenital lung or heart conditions; male gender; age ≤6 months; neuromuscular disorders; and immunodeficiency. Trisomy 21 and cystic fibrosis were also recently identified as possible risk factors.^13^

There are no effective vaccines or specific drugs against RSV. Treatment has remained largely unchanged since the 1960s and is mainly supportive. A number of Cochrane reviews have noted short-term clinical benefit in the use of nebulised adrenaline.^14^ However, meta-analyses on hypertonic saline, bronchodilator and glucocorticoid use have not shown clinical benefit,^15^,^16^ and currently, only supportive management is recommended.

Recently, there has been a tremendous increase in interest and investment within the pharmaceutical sector in vaccine and drug development against RSV. Several exciting developments are being pursued and optimism is high that effective RSV drugs and vaccines are achievable.

## Methods

All clinical trials relating to vaccines, prophylactics or therapeutics against RSV were identified by searching the World Health Organisation International Clinical Trials Registry Platform (WHO ICTRP) (www.who.int/trialsearch/) for the terms ‘RSV’ or ‘respiratory syncytial virus’.^17^ The WHO ICTRP search portal includes all internationally recognised clinical trial databases. See Appendix 1.

Clinical and preclinical information on each agent was identified through PubMed by searching for the drug/vaccine names/clinical trial identifier. MeSH search protocols and free-text searches were used to ensure no relevant data were omitted. Where no peer-reviewed published data was found, additional experimental information was sought directly either from the manufacturer^18^ or from patents describing the specified pharmaceutical.

Pharmaceuticals that underwent clinical trials before 2008 but with no subsequent published information or outcomes were excluded from this review, as we considered that they constituted discontinued drug/vaccine developments.

## Results of search

In January 2015, the WHO ICTRP search portal identified 160 trials registered relating to RSV. Fifty-four trials pre-2008 with no published outcomes were excluded. Of the remaining 106, a further 61 trials were excluded due to irrelevance to the topic, duplication, non-drug trials or trials that did not involve new RSV drugs (Table1). In total, 45 relevant entries relating to the prevention or treatment of RSV were identified. These trials corresponded to 24 different drugs, including 5 prophylactics, 9 therapeutics and 10 vaccines (see Table2 for details). The vaccines, prophylactics and therapeutics currently undergoing clinical trials are described below. This review presents a comprehensive overview of current strategies undergoing clinical development for the clinical management of RSV.

**Table 1:** Results of WHO ICTRP search for relevant clinical trials involving RSV therapeutics, prophylactics or vaccines

Total number of RSV clinical trials identified	160
Trials excluded due to date	54
Records excluded due to other reasons	61
Duplicate entries	11
Non-drug related studies	34
Not relevant to RSV	3
Palivizumab studies	9
Studies not involving new drugs	4
Relevant records included in the review	45

**Table 2:** The most recently registered clinical trials for RSV vaccines, prophylactics and therapeutics since 2008

Drug name	Clinical trial status	Manufacturer/Institution	Experimental approach	Outcome
*Vaccines*				
Live attenuated				
MEDI-559	Phase 1/2a	MedImmune LLC	Live attenuated	Increased rate of LRTIs in vaccine recipients, further study ongoing
RSV LID ΔM2-2	Phase 1	NIAID	Recombinant live attenuated RSV	Estimated completion May 2016
RSV cps2, Lot RSV#005A	Phase 1	MedImmune, NIAID	Recombinant live Attenuated RSV	Estimated completion May 2015
Chimeric/vectored				
RSV 001	Phase 1	Okairos	Adenovirus vector and an MVA vector encoding RSV antigens	Commenced May 2013
				Outcome awaited
MEDI-534	Phase I	MedImmune LLC	Chimeric/vectored	Genetic variants within vaccine detected. Ongoing research.
Nanoparticle				
RSV F nanoparticle vaccine	Phase 2	Novavax	Recombinant RSV F protein particle	Commenced October 2013 Outcome awaited
Subunit				
MEDI 7510	Phase 1a	MedImmune LLC	RSV sF antigen + synthetic glucopyranosyl lipid A adjuvant	Estimated completion July 2015
RSV F subunit vaccine	Phase 1	Novartis vaccines	F subunit vaccine	Estimated completion September 2016
GSK3003891A	Phase 1	GlaxoSmithKline (GSK) Biologicals	Prefusion F subunit vaccine	Estimated completion March 2015
*Prophylactics*				
Motavizumab (Numax/MEDI-524)	Phase 3 (completed 2008)	MedImmune	Affinity-matured palivizumab	Increased type I hypersensitivity.
				No FDA approval
MEDI 557	Phase 1	MedImmune	Recombinant human monoclonal antibody	Completed May 2013. Data not available
MEDI 8897	Phase 1b/2a	MedImmune	Human RSV monoclonal antibody	Estimated completion December 2014
RI-001	Phase 2	ADMA biologics Inc	Polyclonal immune globulin. Delivered intravenously	Completed May 2010
REGN2222	Phase 3	Regeneron pharmaceuticals	Human monoclonal anti-RSV F antibody	Estimated completion date February 2015
*Therapeutics*				
GS-5806	Phase 2b	Gilead	RSV entry inhibitor	Estimated completion June 2015
ALN-RSV01	Phase 2b (completed May 2012)	Ablynx	siRNA targeting the N protein	Reduced bronchiolitis obliterans post-RSV infection in lung transplant recipients.
ALS-008176	Phase 2a	Alios biopharma Inc.	Nucleoside analogue	Estimated completion August 2014
Danirixin	Phase 1	GlaxoSmithKline	CXCR2 antagonist.	Estimated completion July 2014
			Inhibition of neutrophil activation	
RV568	Phase 1	Respivert Ltd	Kinase inhibitor	Completed 2011. Data not available
MDT-637	Phase 1	MicroDose Therapeutx, Inc	Fusion inhibitor	Commenced 2013. Data not available
ALX-0171	Phase 1	Ablynx	Nanobody	Commenced 2014
				Data not available

### Current state of advancement of vaccine, prophylactic and therapeutic options for RSV in clinical development

#### Vaccines

No RSV vaccine has been licensed to date. Enhanced disease following natural RSV infection in the wake of the infamous formalin-inactivated alum-adjuvanted RSV vaccine (FI-RSV)^19^ effectively led to a moratorium on the study of inactivated or subunit RSV vaccine candidates in RSV-naïve infants. Since then, further vaccine development has been impeded by the need to create robust immune responses without causing vaccine-enhanced illness.^20^ As peak incidence of severe disease occurs between 6 weeks and 6 months, a RSV vaccine would ideally be administered within the 1st month of life. However, immune immaturity and the presence of maternal antibodies that may negatively impact on vaccine effectiveness are difficult hurdles to overcome in infant vaccine development. Despite these considerable barriers and historical setbacks, there is evidence to suggest that a RSV vaccine is feasible. First, while RSV infection occurs repeatedly throughout life, the frequency of severe disease decreases in second and subsequent infections.^21^,^22^ Furthermore, the monoclonal antibody (MAb) palivizumab is associated with protection against hospitalisation secondary to RSV infection. Finally, an inverse correlation between maternal antibody virus neutralisation (VN) titres and protection against hospitalisation was reported.^23^,^24^

Four potential target populations for RSV vaccines exist, namely young infants, older children, pregnant women and the elderly (>65 years). The most advanced current strategies for RSV vaccine development are live attenuated RSV vaccine strains and recombinant viral vectors expressing RSV antigens. All vaccines currently in clinical trials are detailed in Table2.

Live attenuated RSV vaccines have been studied for several decades. Historically, these vaccines were produced by a combination of chemical mutagenesis and serial passage at progressively lower temperatures. Cold passaged, temperature-sensitive (*cpts*) vaccines were created with the rationale of restricting viral replication to nasal passages rather than the warmer lower airways, thereby preventing LRTI. Promising data regarding this vaccine strategy came from phase 1 testing of the *cpts*-248/404 vaccine in infants aged 1–2 months.^25^ The study reported virus replication in most recipients (19/22) following the first vaccine dose, but no replication in the vast majority of infants (17/19) following a second dose 1 month later. This demonstrated that vaccine-induced immunity is possible in this age group. However, unacceptable upper respiratory tract congestion precluded further development of this vaccine. Reassuringly, this and other recent studies of live attenuated vaccines found no evidence of FI-RSV-like enhanced disease.^26^

Unfortunately, this approach to RSV vaccines has suffered many setbacks, with major difficulties in balancing vaccine attenuation and immunogenicity. Indeed, early attempts with under-attenuated vaccines were associated with higher rates of nasal congestion, fever, LRTI, cough and otitis media.^25^,^27^,^28^ Conversely, over-attenuated vaccines in RSV-naïve children were associated with greatly reduced infectivity and concomitant reduced nasal cytokine levels, suggesting that they were unsuitable vaccine candidates.^29^,^30^

Reverse genetics technology for RSV, which enables modulation of the viral genome, facilitated the introduction of combinations of specific attenuating mutations to develop new vaccine candidates, for example MEDI-559 or RSVΔNS2Δ1313/1314L. There are four live attenuated RSV vaccines that have recently completed or are currently in phase 1/2a clinical trials. Data from these trials are expected in 2015 and/or 2016.

Following the initial promise of the *cpts*-248/404 RSV vaccine in infants, the virus was further mutated in an effort to improve its attenuation in view of adverse effects. Preclinical studies identified the attenuating effect of deletion of the SH gene and introduction of a temperature-sensitive mutation in the L gene (N1321). The resultant live attenuated RSV vaccine candidate was designated rA2cp248/404/1030/ΔSH.^30^,^31^ The vaccine was well tolerated in RSV-naïve infants, but induced limited virus neutralising (VN) antibody responses in infants under 6 months and demonstrated genetic instability in 30% of isolates recovered following vaccination.^31^ MEDI-559, a derivative of rA2cp248/404/1030/ΔSH, was subsequently engineered with further codon changes at the 248 (L protein codon 831) and 1030 (L protein codon 1321) attenuating mutation sites. A study in RSV-seronegative infants aged 5–24 months demonstrated significant induction of RSV VN antibodies in 59% of MEDI-559 vaccine recipients. However, genetic instability in its attenuating mutations was evident. More worryingly, although numbers were low, there was an increased rate of medically attended LRTIs, including wild-type RSV infections, in the vaccine versus placebo cohorts during follow-up.^32^–^34^

In an effort to produce a more stable vaccine candidate, RSV cps2 Lot RSV#005A was engineered from MEDI-559 with five nucleotide changes (248 mutation, position L831: a10990g; compensatory mutation, position S1313: a12434t/g12435c/c12436a; 1030 mutation: N1321K, t12460a) known to confer genetic stability to the attenuating mutations.^35^ This vaccine is currently undergoing phase 1 clinical trials with an estimated completion date of May 2015.

A second vaccine candidate was derived from MEDI-559 with a deletion of the codon at position 1313, instead of the S1313 mutation described above for RSV cps2 Lot RSV#005A. This candidate also included deletion of the NS2 gene and an amino acid change at position 1314 in the L protein (I1314L) and was designated RSVΔNSΔ1313/1314L. Preclinical studies showed that this candidate replicates at 37°C.^33^ It had comparable viral shedding in nasal washings to MEDI-559 in chimpanzees, indicating similar levels of attenuation.^27^ This vaccine also induced similar or higher serum VN antibodies in juvenile chimpanzees compared to both MEDI-559 and the RSV cps-2.^33^ A phase 1 clinical trial of RSVΔNS2Δ1313/1314L is underway, designed to determine its clinical safety and genetic stability profiles.

Additional non-essential gene deletion mutants were also explored to produce live attenuated vaccines. MEDI ΔM2-2 vaccine has a deletion of the M2-2 gene. Recombinant viruses with M2-2 gene deletion were shown *in vitro* to have decreased RNA replication, attenuated virus growth kinetics and concomitant increases in F and G protein expression.^36^ As F and G proteins are the principal RSV vaccine targets, the combination of growth attenuation and increased F and G protein expression render this vaccine candidate of considerable interest. MEDI ΔM2-2 is currently undergoing a phase 1 clinical trial for safety and immunogenicity in adults, seropositive children and seronegative infants.

MEDI-534 is a recombinant chimeric bovine/human parainfluenza virus type 3 (b/hPIV3), with a genome composed of the hPIV3 HN and F genes, the bPIV3 N, P, M and L genes, and the RSV F gene. The RSV F gene was inserted as an extra-transcription unit between the b/hPIV3 N and P genes.^37^ As such, MEDI-534 constitutes a potential bivalent vaccine against both hPIV3 and RSV. Following translation, the precursor RSV F_0_ is proteolytically cleaved by furin-like proteases to F_1_ and F_2_ subunits, which subsequently form F_1_/F_2_ heterodimers linked by disulphide bridges to produce mature F.^38^ Infection of Vero cells with MEDI-534 or wild-type RSV A2 resulted in similar expression of RSV F_1_ protein, indicating efficient RSV F protein expression. However, altered RSV F protein cleavage following MEDI-534 infection was also evident, although this did not appear to compromise its vaccine potential. Indeed, MEDI-534 induced protective immunity in Syrian golden hamsters against challenge with RSV A2.^37^ However, testing of the vaccine in RSV-seronegative infants revealed genetic variation of MEDI-534 recovered in nasal washes and a higher rate of reported runny/stuffy nose in all vaccine recipients versus placebo within 14 days of vaccination. The genetic mutations included the introduction of premature stop codons within the RSV F open reading frame, consistent with loss of RSV F protein expression. Subsequent *in vitro* testing involving multiple passages in MRC-5 cells also demonstrated acquisition of mutations that resulted in increased viral replication and a reduction in RSV F protein expression.^39^,^40^ These data suggest that *in vitro* testing for genetic stability should be considered prior to clinical development of such RSV vaccines.

Another recent virus-vectored RSV vaccine strategy to enter clinical trials is the prime/boost regime developed by Okairos. It involves simian adenovirus (PanAd3-RSV) and modified vaccinia Ankara (MVA-RSV) virus vectors expressing RSV F, N and M2-1 proteins. Priming is with PanAd3-RSV, either intranasally or intramuscularly (IM), while IM boosting is with either PanAd3-RSV or MVA-RSV. While no peer-reviewed data were found, preclinical data described in patent WO2014005643A1 demonstrated induction of strong protective immunity in mice and cotton rat models of RSV infection. A phase 1 clinical trial in healthy adult volunteers is currently underway and will investigate the safety and immunogenicity of IN or IM PanAd3-RSV priming and IM boosting with either PanAd3-RSV or MVA-RSV.

A number of subunit vaccine candidates are currently under clinical investigation. These vaccines are based on the RSV F protein. The Novavax RSV F nanoparticle vaccine was engineered using the baclofen/sf9 insect cell system to produce post-fusion F protein.^41^ Cotton rats immunised with the nanoparticles demonstrated strong protective efficacy against RSV challenge. In phase 1 clinical trials in adults, the vaccine was well tolerated and induced 4- to 20-fold increases in anti-RSV F IgG in vaccine versus placebo recipients.^41^–^43^ Clinical trials to investigate safety and immunogenicity following IM administration have recently begun in seropositive infants (24–72 months) (phase 1), adults >60 years (phase 2) and women of child-bearing age (phase 2).

MEDI-7510 is a subunit vaccine containing post-fusion RSV F formulated with the synthetic TLR-4 agonist glucopyranosyl lipid A adjuvant. Glucopyranosyl lipid A is known to enhance the magnitude of antibody responses to influenza vaccines.^44^ A phase 1b study is currently recruiting healthy adults >60 years to study safety and immunogenicity following IM administration.

Novartis are developing a subunit vaccine composed of post-fusion RSV F trimers adsorbed to aluminium hydroxide. It induced evidence of serum VN activity and strong protective efficacy in a cotton rat model.^45^ A phase 1 clinical trial of Novartis vaccine in healthy adults, to assess safety and immunogenicity following IM administration, is due for completion in 2016.

Recent structural data indicated that RSV F adopts two conformations, the pre- and post-fusion configurations.^46^ RSV subunit vaccines described above are based on the post-fusion conformation. However, most RSV neutralising antibodies in human sera following RSV infection are directed towards the pre-fusion configuration, suggesting that vaccines based on post-fusion RSV F may be suboptimal.^47^ Accordingly, a number of pre-fusion F candidates are in preclinical development, including the NIH pre-fusion RSV F vaccine, which induced enhanced serum VN titres in mice and non-human primates relative to post-fusion F antigen.^46^ To our knowledge, the only pre-fusion F antigen vaccine currently in clinical trials is GSK3003891A. Patent no. WO2010149745 reported induction of VN antibodies and protection against RSV challenge in a mouse model of RSV infection following IM immunisation with prefusion F in various formulations.^48^ Phase 1 studies of these vaccine formulations are currently underway in healthy adults to assess safety, reactogenicity and immunogenicity.

Despite the increasing variety of RSV vaccine strategies currently under investigation, it is clear from the number of RSV vaccine candidates failing to progress beyond phase 1/2a trials that a number of challenges remain. Historically, there has been a considerable disconnect between excellent efficacy in animal models and limited or no clinical effectiveness for RSV vaccines. This might be explained in part by the capacity of RSV proteins (e.g. F, G, NS1, NS2), to modulate immune responses following infection of humans.^49^,^50^ Therefore, the possibility remains that these RSV vaccine strategies may result in compromised or poorly protective immune responses in humans, as is evident following natural RSV infection. In our opinion, detailed understanding of human immune responses to RSV infection and/or vaccination would greatly help RSV vaccine development.

#### Prophylactics

RespiGam (RSV immune globulin IV) was the first RSV-specific drug ever approved. It consisted of human polyclonal serum screened for high RSV VN activity and was transfused over several hours in relatively large volumes to infant recipients at high risk of developing severe RSV-LRTI (prematurity, bronchopulmonary dysplasia or chronic lung disease).^51^ In parallel with RespiGam, a humanised MAb (palivizumab) was developed and FDA-approved, and has since superseded the use of RespiGam due to increased RSV specificity and a simpler administration protocol. Palivizumab recognises antigenic site 2 on RSV F and is injected IM each month (15 mg/kg) throughout the annual RSV season. It reduced RSV-related hospitalisation rates by 45–55% in a cohort of infants that were classed as high risk for developing severe RSV-LRTI.^52^ Although palivizumab appears to provide some protection against severe RSV-related disease, the cost-benefit ratio is such as to restrict the use to high-risk infants.^53^ Motavizumab, an affinity-matured derivative of palivizumab with a 20-fold increase in VN activity, was found to be more efficient than palivizumab at preventing severe RSV-related disease in high-risk infants.^54^ Unfortunately, it also caused hypersensitivity reactions in some infant recipients and consequently failed to obtain FDA approval.^55^

A major problem associated with palivizumab is the requirement for repeated monthly injections. To help overcome this, MedImmune developed third-generation RSV-specific YTE mutant (amino acid substitutions M252Y/S254T/T256E) MAbs, including MEDI-557 and MEDI-8897, derived from motavizumab and D25, respectively. D25 is a previously described anti-RSV F MAb isolated directly from human B cells.^56^ The YTE mutations confer extended half-lives.^57^ The goal was to develop a pharmaceutical requiring a single annual injection. However, there are currently no published data on either MAb, although clinical trials in healthy adults or preterm infants were recently undertaken (Table2).

REGN2222 a fully human MAb directed against RSV F was derived from RSV-F-immunised transgenic mice expressing human immunoglobulin germ line sequences. This is the only RSV-specific drug to reach phase 3 clinical trials since motavizumab, for which preterm infants are currently being recruited. Although we were unable to locate peer-reviewed data, patent US20140271653 reported a 15- to 17-fold greater *in vitro* VN activity against RSV A2 and superior prophylaxis in a cotton rat model of RSV infection compared with palivizumab.^58^

#### Therapeutics

Recent efforts to develop RSV antiviral drugs have focused primarily on fusion inhibitors or virus gene silencing. Ribavirin, a nucleoside analogue inhibitor of viral RNA synthesis, is licensed for use in haematopoietic transplant recipients with RSV-LRTI. Recent data suggested it may play a role in reducing mortality in this patient cohort.^59^ However, its efficacy and safety profile remains controversial due to inconclusive evidence from small, under-powered studies.^60^,^61^

A novel RSV fusion inhibitor, GS-5806, efficiently neutralised a large panel of RSV clinical strains *in vitro*.^62^ Studies in cotton rats suggested a trend towards a dose-dependent reduction in lung viral titres following intraperitoneal administration 1 hour after RSV challenge. In an exciting development, a RSV strain Memphis 37 challenge trial in healthy adults with low serum RSV VN activity showed reduced mucus production, clinical severity scores, and mean peak viral loads in nasal washes following treatment.^63^ It is noteworthy, however, that GS-5806 was administered orally following RSV detection but before symptoms developed.^63^ The excitement generated by these data, therefore, must be tempered by the fact that the experimental protocol poorly reflects clinical reality, in which symptomatic infections will be evident before drug administration. However, this concern may be resolved in a forthcoming trial in infants hospitalised with RSV (due for completion by June 2015).

RNA interference (RNAi) is a process that targets specific mRNAs for degradation, thereby abrogating expression of the encoded proteins. Synthetic RNAi derivatives, such as short interfering RNAs (siRNA), were shown to be effective therapeutics against several genetic diseases, cancers and viral infections.^64^–^67^ ALN-RSV01 is an siRNA directed against the RSV nucleocapsid (N) protein.^68^ A clinical trial in otherwise healthy RSV-infected adults showed a trend towards reduced mean viral titres and clinical severity scores, although neither reached statistical significance.^69^ ALN-RSV01 has also been tested in a small cohort of RSV-infected lung transplant recipients (*n* = 24; ALN-RSV01 = 16, placebo = 8). The incidence of bronchiolitis obliterans syndrome was greatly reduced, with only 6·3% of patients receiving ALN-RSV01 developing new or progressive bronchiolitis obliterans, compared with 50% of the placebo group.^70^ No data have been published on ALN-RSV01 since 2011, but development appears to be ongoing.

In a similar RSV Memphis 37 human challenge model as described above, the nucleoside analogue, ALS-008176, was shown to significantly reduce viral load and accelerate viral clearance, with a trend towards reduced clinical severity.^71^ However, the data remain to be peer-reviewed. As was reported for GS-5806, this drug was administered shortly after a positive PCR test for RSV in nasal washes. As such, the time at which the drug was administered may reduce the clinical significance of these data. A phase 2a safety study with ALS-008176 in infants hospitalised with RSV is ongoing, and preliminary efficacy data are also expected from this study.

MDT-637 is a fusion inhibitor delivered as an inhaled dry powder. Preclinical data demonstrated that the drug can be dispersed through the upper and lower airways using this delivery system.^72^ A proof-of-concept efficacy trial using the RSV Memphis 37 human challenge model was initiated in 2013. No data have yet been published, but proprietary information suggested this drug is more potent than ribavirin.

Single-domain camelid-derived antibodies, or nanobodies, are antibody fragments that retain the antigen-binding ability of the heavy chain antibody.^73^ ALX-0171 is a trivalent RSV-F-specific nanobody with potent VN activity.^74^ Its therapeutic potential in a neonatal lamb model of RSV infection was recently reported at an international RSV conference.^75^ Treatment initiation by inhalation post-RSV challenge, even following appearance of symptoms, resulted in a dramatic decline in cultivatable virus and reduced lung viral antigen expression, lung viral lesions and histological changes.^75^,^76^ ALX-0171 treatment also exerted a positive effect on clinical parameters (e.g. respiratory rate, wheeze, temperature) and was well tolerated. It was reported to be safe in phase 1 safety and pharmacokinetic studies in adults and a phase 1/2a study in RSV-infected hospitalised infants aged 5–24 months is ongoing^77^ (Table2).

Inflammatory responses to RSV infection are thought to be major components of RSV pathogenesis. In particular, neutrophil infiltration to the lungs is associated with severe disease.^78^ Targeting inflammatory responses is, therefore, being pursued as a strategy for treating RSV-associated disease. Danirixin (GSK1325756) is a reversible CXCR2 antagonist originally developed as an anti-inflammatory agent for disorders associated with neutrophil accumulation, such as chronic obstructive pulmonary disease.^79^ As neutrophil infiltration is an important component of RSV pathogenesis, a clinical trial was initiated to determine the capacity of danirixin to inhibit neutrophil activation in RSV-infected infants. Specifically, the relative capacity of escalating doses of danirixin to block expression of the activation marker CD11b on CXCL1-stimulated peripheral blood neutrophils derived from RSV-infected children <2 years of age or healthy adults was examined. Although study completion was expected in July 2014, data remain to be published.

Neutrophil responses were also targeted by the narrow spectrum kinase inhibitor, RV568, which was initially developed for the treatment of inflammatory diseases, such as rheumatoid arthritis. It was recently repositioned for investigation against RSV infection.^80^ In a RSV Memphis 37 human challenge trial, subjects treated intranasally bis in die with RV568 from 24 hours post-challenge showed a reduction in IL-8 levels in nasal washes relative to untreated controls.^81^ Although evidence suggests that increased IL-8 levels are associated with more severe RSV disease and IL-8 is a neutrophil chemotactic, this study showed no effect on clinical severity.^82^

Many of the therapeutic studies described above used viral load as the primary outcome. However, there is conflicting evidence as to whether viral load correlates with severe disease in humans. Some studies demonstrated a correlation between increased viral load and disease severity,^83^,^84^ but another found no correlation.^85^ A combination of host and viral factors are likely to contribute to the overall pathogenesis of RSV.^86^ As such, a combination of antiviral and anti-inflammatory agents may be necessary for a successful therapeutic. In this regard, identification and a comprehensive understanding of host factors implicated in RSV pathogenesis will greatly facilitate the design and outcomes of clinical trials involving RSV drugs.

## Conclusions

Respiratory syncytial virus is the most important cause of LRTI in young children worldwide and continues to be a major unmet medical need for most infants. Despite the generation of extensive information regarding RSV pathogenesis in animal and cell infection models, the mechanisms of RSV pathogenesis in humans remain elusive. With the exception of palivizumab and RespiGam, no other successful prophylactics are licensed for use. Furthermore, no RSV-specific therapeutic or vaccine has been licensed. Encouragingly, there has been an increase recently in RSV vaccines and pharmaceuticals entering clinical trials. These products were often derived from a greater understanding of RSV attenuation and structural biology, and the application of novel technologies, such as nanobodies, nanoparticles, siRNA and small molecule inhibitors. However, as RSV pathogenesis is thought to be immune mediated and primary infection does not prevent re-infection, a deeper fundamental understanding of RSV disease mechanisms and immune modulation in humans is likely to be necessary for successful clinical development of vaccines, prophylactics or therapeutics.

## Appendix 1 National clinical trials registries included in the WHO registry of clinical trials

- Australian New Zealand Clinical Trials Registry, last data file imported on 8 December 2014.
- Chinese Clinical Trial Registry, last data file imported on 8 December 2014.
- ClinicalTrials.gov, last data file imported on 8 December 2014.
- EU Clinical Trials Register, last data file imported on 8 December 2014.
- ISRCTN, last data file imported on 8 December 2014.
- The Netherlands National Trial Register, last data file imported on 9 December 2014.
- Brazilian Clinical Trials Registry (ReBec), last data file imported on 9 December 2014.
- Clinical Trials Registry – India, last data file imported on 9 December 2014.
- Clinical Research Information Service – Republic of Korea, last data file imported on 9 December 2014.
- Cuban Public Registry of Clinical Trials, last data file imported on 9 December 2014.
- German Clinical Trials Register, last data file imported on 9 December 2014.
- Iranian Registry of Clinical Trials, last data file imported on 10 November 2014.
- Japan Primary Registries Network, last data file imported on 9 December 2014.
- Pan African Clinical Trial Registry, last data file imported on 9 December 2014.
- Sri Lanka Clinical Trials Registry, last data file imported on 9 December 2014.
- Thai Clinical Trials Register (TCTR), last data file imported on 9 December 2014.
